# Factors contributing to medication non-adherence in a hypertensive Saudi population: a literature review

**DOI:** 10.25122/jml-2025-0154

**Published:** 2025-12

**Authors:** Ahmed Alenazi, Wejdan Alhajri, Waroud Alruwili, Sokaina Alnowiser, Ohud Alsudyyes, Sara Hijazi, Ibtisam Alotaibi, Manal Albukami, Madhwi Aldhfere, Shima Daak, Lujain Alhomaid, Mona Alshamery

**Affiliations:** 1 Pharmaceutical Care Department, Imam Abdulrahman Bin Faisal Hospital, Ministry of National Guard Health Affairs (MNGHA), Dammam, Saudi Arabia; 2 King Abdullah International Medical Research Center, Riyadh, Saudi Arabia; 3 King Saud bin Abdulaziz University for Health Sciences, Riyadh, Saudi Arabia; 4 Department of Pharmaceutical Services, King Fahad Military Medical Complex (KFMMC), Dhahran, Saudi Arabia; 5 College of Pharmacy, Almaarefa University, Riyadh, Saudi Arabia; 6 Research and Development, Lean Business Services, Riyadh, Saudi Arabia; 7 Department of Pharmaceutical Care, Al Mouwasat Hospital, Khobar, Saudi Arabia; 8 Department of Pharmaceutical Services, Prince Sultan Military Medical City, Riyadh, Saudi Arabia; 9 College of Pharmacy, Jazan University, Jazan, Saudi Arabia; 10 College of Pharmacy, King Saud University, Riyadh, Saudi Arabia; 11 Al-Dawaa Medical Services, Dammam, Saudi Arabia

**Keywords:** medication compliance, adherence, hypertension, Saudi

## Abstract

Hypertension is a leading non-communicable disease both globally and in Saudi Arabia. Poor adherence to antihypertensive medications remains a major challenge in managing this condition, contributing to higher morbidity, mortality, and healthcare costs. This study aimed to review and synthesize the literature on factors contributing to medication nonadherence among hypertensive patients in Saudi Arabia. A structured literature review was conducted using the PubMed and Scopus databases. Inclusion criteria include English-language studies conducted in Saudi Arabia from 2010 to 2024 involving hypertensive adult Saudi patients. A total of 84 studies were identified. Eleven studies met the final inclusion criteria. All were cross-sectional studies using different validated adherence assessment tools. Adherence rates varied widely, from 33% to 86.1%, influenced by the choice of measurement tool and regional differences. Higher adherence was associated with older age, female gender, higher education level, being married, and higher income. Psychosocial and behavioral factors, including beliefs about the necessity of medication, perceived social support, and strong physician-patient relationships, positively influenced adherence. One study using the Health Belief Model (HBM) found that perceived severity, susceptibility, benefits, and cues to action significantly affected adherence behaviors. Medication adherence among hypertensive patients in the Saudi population is influenced by a complex relationship of demographic, psychosocial, and healthcare-related factors. Addressing these elements through tailored interventions and appropriate assessment tools may enhance hypertension management outcomes.

## INTRODUCTION

Hypertension is one of the most prevalent non-communicable diseases, currently affecting nearly 1.3 billion people worldwide. It is a major risk factor for cardiovascular diseases, stroke, and kidney failure. The number is expected to rise to 1.5 billion by 2025 [1-3]. In Saudi Arabia, the prevalence of hypertension varies according to several factors, including age, gender, education level, region, occupation, and access to healthcare services. Reported prevalence rates range from 22% to 25.5% [4,5], while the estimated average across Middle Eastern countries is around 29.5% [3].

**Table 1:** Factors associated with non-adherence to antihypertensive medication in the Saudi population

Categories	Factors
Patient-related factors [19,20-23,25-29]	Demographic factors: age, ethnicity, gender, education, marital status Psychosocial factors: beliefs, motivation, attitude Patient-prescriber relationship Health literacy Patient knowledge Physical difficulties Tobacco use or alcohol intake Forgetfulness
Therapy-related factors [19,24]	Route of administration Treatment complexity Length of the treatment intake Medication side effects Degree of behavioral change required Taste of the medication Requirements for drug storage
Healthcare system factors [24]	Lack of accessibility Long waiting time Difficulty in getting prescriptions filled Unpleasant clinic visits
Social and economic factors [25,29,35,37]	Inability to take time off work Cost of the treatment Unaffordable treatment Lack of social support
Disease factors [19-21,24,32,35]	Disease symptoms Severity of the disease

The growing burden of hypertension in Saudi Arabia is primarily attributed to an aging population and the increasing prevalence of lifestyle-related risk factors, including unhealthy diets and physical inactivity. In response, the Saudi government has prioritized health improvement through its Vision 2030 initiative, particularly the Health Sector Transformation Program, which aims to increase average life expectancy to 80 years by strengthening healthcare quality, promoting preventive care, and encouraging healthier lifestyles. Despite these efforts, poor medication adherence remains a major challenge in the effective management of hypertension, contributing significantly to its role as one of the leading causes of death and disability in the country [6-8].

Non-adherence to medication and other aspects of the treatment plan can adversely affect treatment outcomes, leading to additional and unnecessary tests, dosage adjustments, treatment plan changes, revisits to the emergency department, or hospitalization, which ultimately result in increased health care service costs, morbidity, and mortality [9,10]. Non-adherence to medication can be intentional or unintentional on the part of the patient. Intentional non-adherence refers to a patient’s purposeful decision not to follow the treatment plan, often influenced by their motivations or beliefs. In contrast, unintentional non-adherence is typically the result of a lack of the capacity, resources, or understanding required to take medications as prescribed [11].

The terms adherence and compliance are globally debated and often used interchangeably in the healthcare field [12]. At the World Health Organization (WHO) Adherence meeting in June 2001, adherence was defined as “the extent to which the patient follows the medical instructions” [13]. According to the National Health Service (NHS), adherence refers to “the extent to which the patient’s behavior matches agreed recommendations from the prescriber”, whereas compliance is defined as “the extent to which the patient’s behavior matches the prescriber’s recommendations” [12]. In this review, the compliance and adherence terms will be used interchangeably.

**Table 2:** Validated tools for evaluating patient medication adherence

Scale	Items	Domains	Focus	Scoring	Strengths	Limitations
Hill-Bone Medication Adherence Scale (HBMAS)	9	Medication-taking behavior	Hypertension medication adherence	Likert (1–4)	Simple; validated in HTN settings	Limited to HTN; cultural adaptation needed
Malaysian Medication Adherence Score (MALMAS)	8	Intentional and unintentional non-adherence	Chronic diseases (Malaysia)	Mixed (Yes/No + Likert)	Culturally tailored; validated	Country-specific; limited generalizability
Morisky Green and Levine adherence scale (MGL)	4	Forgetfulness, stopping when better/worse, carelessness	General adherence	Yes/No (0–4)	Very quick; widely used	Oversimplified answers
Morisky Medication Adherence Scale-4 (MMAS-4)	4	Unintentional & intentional non-adherence	General medication adherence	Yes/No	Easy to administer	Less detailed than MMAS-8
Morisky Medication Adherence Scale-8 (MMAS-8)	8	Unintentional & intentional non-adherence	General medication adherence	Mixed (7 Yes/No + 1 Likert)	Comprehensive; distinguishes behavior types; validated across diseases	Requires permission/licensing; longer to administer

Numerous factors associated with non-adherence have been identified in the literature. These factors may be grouped into categories including patient-related factors, therapy-related factors, healthcare system factors, social and economic factors, and disease factors (Table 1) [14]. The impact of these factors on medication adherence is variable and controversial, with some studies identifying clear factors that enhance medication adherence, while others fail to replicate these findings.

In connection with this, numerous validated instruments have been created to assess medication adherence from various clinical viewpoints, including the Hill-Bone Medication Adherence Scale (HBMAS) [15], the Malaysian Medication Adherence Score (MALMAS) [16], the Morisky Green and Levine scale (MGL), the Morisky Medication Adherence Scale-4 (MMAS-4), and the Morisky Medication Adherence Scale-8 (MMAS-8) (Table 2) [17,18]. The selection of an appropriate scale should be influenced by the intended population, the clinical setting, and the level of behavioral insight required.

## MATERIAL AND METHODS

### Study design and search

The review was conducted using PubMed and Scopus to identify studies published between 2010 and 2024. The search strategy combined relevant keywords and MeSH terms to collect research on medication adherence among hypertensive patients in Saudi Arabia. The main search structure utilized was: (hypertension OR high blood pressure) AND (adherence OR compliance) AND (Saudi Arabia OR KSA). In the PubMed search, this was refined using MeSH terms: ("Hypertension"[MeSH] OR "Antihypertensive Agents"[MeSH]) AND ("Medication Adherence"[MeSH] OR "Patient Compliance"[MeSH]) AND "Saudi Arabia"[MeSH]. The search was confined to the English language and human studies only.

**Figure 1:**
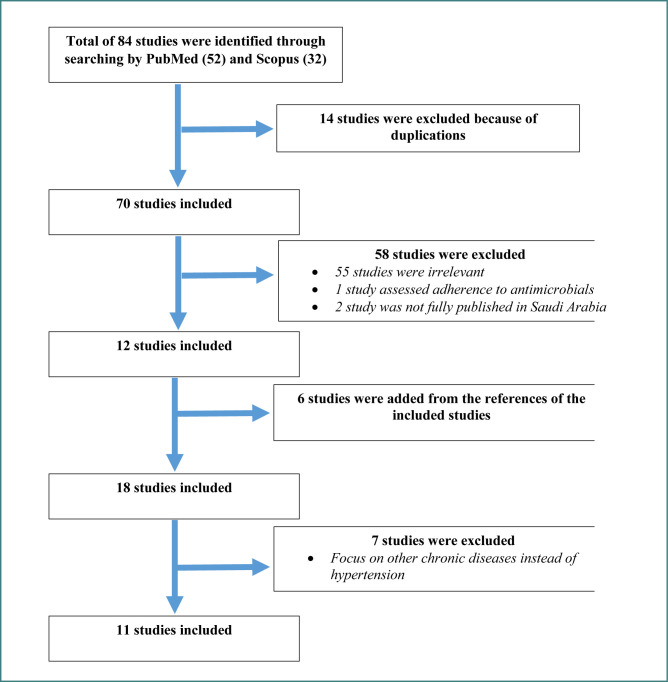
Flow diagram of the study selection process

### Eligibility criteria

**Inclusion criteria:** studies published in the English language between 2010 and 2024, involving adult Saudi patients (aged ≥ 18 years) with hypertension.

**Exclusion criteria:** studies that included non-Saudi populations and did not focus on factors contributing to antihypertensive medication non-adherence.

### Quality assessment

The quality of the included cross-sectional studies was evaluated independently by two reviewers using the Joanna Briggs Institute (JBI) critical appraisal checklist for analytical cross-sectional studies. The checklist covers key domains, including sample representativeness, methods of participant recruitment, adequacy of sample size, objectivity of measurements, and appropriateness of statistical analyses. Any differences in judgment were discussed and resolved by consensus.

### Findings

We identified 84 records through PubMed (*n* = 52) and Scopus (*n* = 32). After removing 14 duplicates, 70 studies remained for title and abstract screening, of which 58 did not meet the inclusion criteria due to being irrelevant or not fully published in Saudi Arabia. We reviewed the full texts of the remaining 12 studies and, through manual reference checking, identified 6 additional relevant articles, bringing the total to 18 studies for full review. After assessing these in detail, 7 were excluded because they focused on other chronic diseases. In the end, 11 studies met all criteria and were included in this review (Figure 1). All included studies were cross-sectional and involved Saudi adults with hypertension, with sample sizes ranging from 144 to 549 participants.

### Study selection

Studies were selected after screening the titles and abstracts to ensure that they met the inclusion criteria. The studies were screened for the definition of adherence, the level and prevalence of adherence, setting, population, sample size, methodology, data collection, and the instruments used to measure adherence.

## RESULTS

A review of 11 cross-sectional studies conducted in multiple regions of Saudi Arabia, involving 4,194 participants, was performed to investigate adherence to antihypertensive medications (Table 3). The studies used recognized adherence evaluation tools, such as the Hill-Bone Medication Adherence Scale, the Morisky Medication Adherence Scale (MMAS-4 and MMAS-8), the Morisky-Green and Levine (MGL) scale, and the Malaysian Medication Adherence Score (MALMAS-8) [19-29]. Overall, the studies were of moderate quality. Most of these studies clearly described their study populations and used validated tools to assess adherence, which is a consistent strength across the included literature.

**Table 3:** Characteristics of the included studies on antihypertensive medication adherence in Saudi Arabia

Author(s) (Year)	Region of KSA	Study design	Number of participants	Adherence measurement tool	Key non-adherence factors identified (summarized)
Alsolami *et al*. (2015) [19]	Not Specified	Cross-sectional	200	MMAS-8	Low belief in necessity, high concern about side effects, weak physician-patient relationship
Elbur (2015) [20]	Taif	Cross-sectional	200	MGL	Younger age, lower education level, absence of comorbidities
Abdelhalim *et al*. (2019) [21]	Dammam	Cross-sectional	303	HBMAS	Younger age, male gender, lack of reminders/cues to action
Algabbani *et al*. (2020) [24]	Riyadh	Cross-sectional	340	MMAS-8	Polypharmacy, frequent clinic visits
Alsofyan *et al*. (2022) [25]	Taif	Cross-sectional	231	MMAS-4	Female gender, older age, being married, higher education
Thirunavukkarasu *et al*. (2022) [22]	Abha	Cross-sectional	400	MMAS-8	Being unmarried, low monthly income
Innab *et al*. (2023) [23]	Multiple	Cross-sectional	549	MMAS-8	Younger age, lower patient activation
Fallatah *et al*. (2023) [26]	Jeddah	Cross-sectional	384	MMAS-8	Younger age, lower education
Alfhaid *et al*. (2024) [27]	Majmaah	Cross-sectional	144	MMAS-8	Male gender, low adherence overall
ALruwaili (2024) [28]	Aljouf	Cross-sectional	303	HBMAS	Employment status (private sector linked to higher adherence)
Almaghamsi *et al*. (2024) [29]	Jeddah	Cross-sectional	500	MALMAS-8	Lower education, lack of social support, being a government employee

The reported medication adherence levels ranged widely. Overall, high adherence rates ranged from 34.3% to 86.1%, with non-adherence rates up to 67%. Alfhaid *et al.* in Majmaah reported the lowest adherence at 33% using the MMAS-8 scale [27], while Alsofyan *et al.* in Taif had the highest adherence rate at 86.1% with the MMAS-4 scale [25]. This inconsistency in reported medication adherence rates is attributed to regional variations and methodological differences, particularly the choice of adherence scale.

Various socio-demographic factors have consistently been correlated with antihypertensive medication adherence. Gender differences were noted, with women generally exhibiting higher adherence rates than men [21,25]. Older individuals, particularly those aged 60 and older, were associated with higher adherence levels [23, 25, 26]. Thirunavukkarasu and Alsofyan’s results showed that marital status appeared to play a meaningful role in adherence, with married patients generally showing better adherence to their antihypertensive medications than non-married patients [22,25]. In addition, Elbur and Almaghamsi showed that patients with higher education qualifications tended to be more adherent to and follow their treatment plans [20,29]. Alsofyan and Almaghamsi found that higher income was connected to better medication adherence [25,29]. Interestingly, Alruwaili’s research showed a significant association between private-sector employment and higher adherence rates [28].

Elbur’s study demonstrated that comorbid conditions had a substantial impact on medication adherence, indicating that patients with these conditions were more likely to adhere to their prescribed treatment regimens [20]. Algabbani *et al*. observed that patients prescribed fewer medications or who attended fewer clinic visits exhibited higher adherence to antihypertensive medications [24].

Psychosocial and behavioral factors were found to significantly affect medication adherence. A strong belief in the necessity of medications and low concerns about potential side effects were associated with improved medication adherence, as noted in Alsolami’s research [19]. In the same study, Alsolami also highlighted that a strong physician-patient relationship was significantly associated with better medication adherence [19]. Social support was also recognized as a key factor, with individuals who perceive higher levels of support showing improved adherence, as demonstrated in Almaghamsi’s study [29]. Abdelhalim *et al.* found that reminders and other cues to action were especially helpful for those who perceived more barriers, suggesting that external prompts can make a real difference when internal motivation is challenged [21].

Abdelhalim *et al*., in a study that implemented the Health Belief Model (HBM), which predicts health/disease risk and preventive behavior [30], identified several psychological constructs as important predictors of medication adherence. Greater perceived severity of hypertension, susceptibility to complications, and perceived advantages of medication were all linked to improved adherence. Notably, a higher perception of treatment barriers was also positively correlated with adherence, suggesting that patients who recognized more challenges might be more driven by external cues such as reminders. This finding emphasizes the role of behavioral signals in navigating perceived obstacles and boosting compliance [19].

## DISCUSSION

This literature review of 11 cross-sectional studies involving 4,194 participants across various regions of Saudi Arabia provides important insights into the multifaceted nature of adherence to antihypertensive medications. Reported adherence levels varied widely, with high adherence rates ranging from 34.3% to 86.1% and non-adherence reaching 67%. This variability is in line with the international literature and appears to be influenced by both patient-related and methodological factors, including the type of adherence measurement tool used.

A key finding is the substantial heterogeneity in adherence rates, partly due to the use of different validated tools (e.g., MMAS-4, MMAS-8, MGL, Hill-Bone, and MALMAS-8). Eventually, variations in measurement tools also affected the observed adherence rates. MMAS-8, which includes a wide range of items and can distinguish between intentional and unintentional non-adherence, in general, provides more comprehensive insights. On the other hand, shorter tools like the MMAS-4 and MGL deliver simpler evaluations that may overlook the complexities of patient behavior. An 86.1 percent adherence rate was the highest achieved with the MMAS-4, while 33 percent was the lowest with the MMAS-8, highlighting how the choice of scoring scale can strongly impact study findings.

The association between socio-demographic factors and medication adherence was consistently highlighted in the studies reviewed. Gender-related results were somewhat conflicting. Some studies found that women were more adherent [21,25-27,31], whereas other studies by Khayyat and Mahmoud reported lower adherence among female patients [32,33]. Furthermore, other studies by Alsolami, Almaghamsi, and Algabbani did not establish any link between sexuality and medication adherence [19, 24, 29]. This aligns with global trends where gender is not a consistent predictor of adherence. The reasons for this gender discrepancy remain unclear. This issue may contribute to differences in health consciousness, health literacy, or cultural aspects of health. However, more research is needed to validate these hypotheses and identify the best approaches to improving medication adherence rates among both men and women with chronic diseases.

A positive finding was observed in older adults. Several studies have reported a significant association between age and improved medication adherence, especially among those over 50 years [21,23,25,26]. This could be due to increased health awareness, the severity of the disease, or the presence of caregivers. This finding is in line with earlier research suggesting that improvements in adherence related to age may be influenced by factors such as assistance with medication management and greater involvement in healthcare systems [34]. Another possible reason is holding different opinions and beliefs about medication adherence, influenced by cultural and socio-economic aspects. Furthermore, other studies have indicated that being younger than 50 years is a risk factor for low medication adherence [19,21-24,29].

Mixed results regarding the relationship between marital status and medication adherence were noticed. A weak association was found between being married and adherence to medications [25, 29, 32]. This was further supported by findings from Thirunavukkarasu *et al.*, who indicated that unmarried patients had significantly lower adherence to antihypertensive medication compared to their married counterparts [22]. A further study by Khayyat *et al*. also confirmed that married patients were more likely to adhere to their medication than others [32]. Conversely, a finding by Alkhamis *et al*. observed that marriage did not significantly help patients in following their medication regimens [35]. However, married individuals generally demonstrated better adherence, likely due to the emotional and logistical support received from their partners [25,29].

Education was positively linked to adherence in several studies. Patients with higher levels of education may have a better understanding of the consequences of uncontrolled hypertension and the benefits of adherence [19,20,26,29]. In support of this, Alsofyan *et al*. reported that patients with low education levels and those who were illiterate showed lower adherence to their medications [25]. Moreover, Abdelhalim *et al.* found that patients who were aware of their treatment plans and knowledgeable about their conditions were more likely to adhere to their medication regimens [21]. It is important to note that, in some cases, highly educated individuals may also question the need for medication due to their greater awareness of potential side effects [19, 29, 36]. Despite this, many studies have shown that higher levels of education were not necessarily associated with medication adherence [24, 27, 28]. This observation is similar to other Saudi studies published earlier [31,37,38].

A significant contradiction in research related to medication adherence occurred when patients were diagnosed with multiple chronic conditions and comorbidities. Our review shows that most of the included studies reported no significant association between comorbidities and medication adherence [22, 24-26]. This may be due to possible comorbidities associated with old age and polypharmacy medications, which possibly increase the chance of forgetting the time of taking the medication. Other possibilities include being careless with their medications or not being accessible most of the time. This finding was also supported by earlier studies by Khayyat and Alkhamis, which showed that patients with multiple comorbidities were less likely to be adherent to medication than patients with a single disease [32,35].

Occupation and monthly income were positively associated with adherence in several studies. Employees in the private sector showed better adherence in the Alruwaili study [28]. However, the Almaghamsi study reported that government employees showed higher medication adherence compared to others [29]. This may reflect both increased health literacy and access to employer-sponsored healthcare benefits. The relationship between income and adherence is complex, and most reviewed studies found no significant association between patients’ monthly income and medication adherence [19,20,22-24,26-28]. Interestingly, Alkhamis *et al*.’s study reported that patients with low monthly income (<5,000 SR) were more likely to have lower medication adherence, whereas those with high monthly income (>10,000 SR) had higher medication adherence [35]. We assume that higher monthly income facilitates better access to healthcare resources, consequently improving adherence. This finding was consistent with a study conducted in Makkah among patients with various chronic diseases, which found that those with a low monthly income (< 6,000 SR) had twice the risk of non-adherence to medications [37]. Non-adherence was also two times detected in patients who purchased their medication at their own expense compared to those who had insurance or governmental coverage [37].

### Future directions

This review points out several critical gaps in the current understanding of antihypertensive medication adherence in Saudi Arabia. To fortify future research, there is a necessity for study designs that go beyond simple cross-sectional snapshots. Longitudinal studies would help monitor adherence trends over time and identify authentic predictors, while qualitative and mixed-methods approaches are vital for revealing the cultural, social, and personal factors that lead to non-adherence—insights that cannot be fully captured by surveys alone. Future initiatives should also transition towards creating and assessing culturally tailored interventions. Examples include faith-aligned strategies to facilitate medication adherence during Ramadan, family-oriented educational programs, and digital health tools tailored to local communication platforms and Arabic dialects. Standardizing adherence measurement with validated instruments such as the MMAS-8, or a culturally adapted equivalent, would enhance comparability across studies and regions. Ultimately, targeted research is necessary for under-explored groups—such as younger men, individuals with limited health literacy, and residents of less-represented regions—to better comprehend their unique challenges and guide the development of focused interventions.

### Limitations

The review presents several limitations that should be recognized. All studies included were cross-sectional, which limits the capacity to determine causal relationships between the identified factors and medication adherence. The majority of studies depended on self-reported adherence measures, which introduces potential recall and social desirability biases that could have inflated adherence estimates. Significant heterogeneity was also noted in the definitions and measurements of adherence, as well as in the characteristics of the study populations, complicating direct comparisons. Key contextual factors—such as access to medications, religious practices, and family-related influences—were inconsistently addressed across studies and may not have been comprehensively captured. Furthermore, excluding non-English publications raises concerns about language bias and may have led to the omission of pertinent findings from Arabic-language sources. Lastly, publication bias cannot be entirely dismissed, as studies that report significant results are more likely to be published and indexed in the utilized databases.

## CONCLUSION

This literature review highlights the significant variability in medication adherence among hypertensive patients in Saudi Arabia, with reported adherence rates ranging from 33% to 86.1%. Regional disparities, measurement tools, and patient-related factors influence the variation. Key contributors to non-adherence include young age, low education, weak social support, and poor physician–patient relationships. The use of the Health Belief Model (HBM) adds a valuable dimension to understanding behavioral influences on adherence. To improve outcomes, healthcare providers must consider culturally sensitive strategies, reinforce patient education, and promote behavioral interventions tailored to individual beliefs and barriers. Implementing standardized tools (e.g., MMAS-8) alongside behavior-based frameworks (e.g., HBM) could improve adherence and enhance patient outcomes in hypertensive care.
